# Haplotype inference in crossbred populations without pedigree information

**DOI:** 10.1186/1297-9686-41-40

**Published:** 2009-08-11

**Authors:** Albart Coster, Henri CM Heuven, Rohan L Fernando, Jack CM Dekkers

**Affiliations:** 1 Animal Breeding and Genomics Centre, Wageningen University, Wageningen, The Netherlands; 2 Clinical Sciences of Companion Animals, Faculty of Veterinary Medicine, Utrecht University, Utrecht, The Netherlands; 3 Department of Animal Science, Center for Integrated Animal Genomics, Iowa State University, Ames, Iowa, USA

## Abstract

**Background:**

Current methods for haplotype inference without pedigree information assume random mating populations. In animal and plant breeding, however, mating is often not random. A particular form of nonrandom mating occurs when parental individuals of opposite sex originate from distinct populations. In animal breeding this is called *crossbreeding *and *hybridization *in plant breeding. In these situations, association between marker and putative gene alleles might differ between the founding populations and origin of alleles should be accounted for in studies which estimate breeding values with marker data. The sequence of alleles from one parent constitutes one haplotype of an individual. Haplotypes thus reveal allele origin in data of crossbred individuals.

**Results:**

We introduce a new method for haplotype inference without pedigree that allows nonrandom mating and that can use genotype data of the parental populations and of a crossbred population. The aim of the method is to estimate line origin of alleles. The method has a Bayesian set up with a Dirichlet Process as prior for the haplotypes in the two parental populations. The basic idea is that only a subset of the complete set of possible haplotypes is present in the population.

**Conclusion:**

Line origin of approximately 95% of the alleles at heterozygous sites was assessed correctly in both simulated and real data. Comparing accuracy of haplotype frequencies inferred with the new algorithm to the accuracy of haplotype frequencies inferred with PHASE, an existing algorithm for haplotype inference, showed that the DP algorithm outperformed PHASE in situations of crossbreeding and that PHASE performed better in situations of random mating.

## Background

In general, marker genotypes of polyploid organisms are *unordered*, i.e. it is unknown to which of the two homologous chromosomes each allele at each marker belongs. The sequence of alleles at adjacent markers on one chromosome is called a *haplotype*; in diploid organisms a genotype consists of two haplotypes. Haplotypes provide information about the cosegregation of chromosomal segments and can be used to identify relatives when pedigree information is unknown. The haplotypes that an individual carries can be determined experimentally but this is expensive [1]. Alternatively, haplotypes can be inferred, either with or without pedigree information.

When pedigree information is available, haplotypes can be inferred using genotype data of relatives (e.g. [2,3]). When pedigree information is not available, haplotypes can be inferred from genotype data of the population (e.g. [4,1-8]).

Stephens et al. [1] used a Bayesian model to obtain a posterior distribution of haplotypes. Their prior distribution for haplotypes approximates a coancestry model by which distinct haplotypes originate from one common haplotype and can differ due to mutations at specific locations. Due to this prior, new haplotypes are likely to be equal or similar to haplotypes that already have been inferred. Stephens and Sheet [8] extended the prior in [1] with a recombination model which explicitly accounts for linkage of loci on the genome. The whole algorithm is implemented in the program PHASE.

The model of Xing et al. [7] is comparable to the model of Stephens et al. [1] in assuming that haplotypes in the population originate from a latent set of ancestral haplotypes. This model uses a Dirichlet Process as prior for the ancestral haplotypes in the population and distinct haplotypes in the population can be associated to one ancestral haplotype due to a mutation rate.

Mentioned methods assume a random mating population where the probability of an ordered genotype is the product of the population frequencies of the two contributing haplotypes [9]. Random mating, however, is rarely accomplished in reality. Departures from Hardy-Weinberg equilibrium that lead to increased heterozygosity complicate haplotype inference, whereas departures that lead to increased homozygosity make haplotype inference easier [1]. A common case of nonrandom mating occurs when parental individuals of opposite sex originate from divergent populations. In animal breeding this is referred to as *crossbreeding *and in plant breeding as *hybridization*. In these applications, selection takes place in the purebred population and crossed offspring are used for production purposes. This allows the breeder to exploit heterosis and reduces the risk of sharing improved genetic material with competitors. Pedigree of crossed individuals is generally not recorded in commercial animal production situations because of logistics and costs [10]. Because of nonrandom mating, haplotypes of commercial crossed individuals can generally not be inferred with the use of existing methods for haplotype inference without pedigree.

During recent years, use of marker information for estimation of breeding values has received ample attention (e. g. [11,10-16]). In general, methods for estimating breeding values with marker data estimate effects the alleles of markers in the data with a specific regression technique and use these effects to calculate breeding values of selection candidates. Direct application of methods for estimating breeding values in crossbreeding situation can be problematic when association phase between markers and QTL differ in the two parental lines, which is increasingly likely when the distance between markers and QTL increases. A secure approach is therefore to estimate separate marker effects for each purebred population separately; this requires knowledge of the line origin of alleles.

Line origin of alleles can be estimated with the use of pedigree information. If pedigree information is not available, line origin of alleles can be estimated based on allele frequencies in the purebred populations, or alternatively, based on haplotype frequencies in the purebred populations. Use of haplotype frequencies can be advantageous to reveal line origin of allele when differences between allele frequencies in both lines are relatively small.

In this article, we introduce a new method for inferring haplotypes in crossbred situations without pedigree information. The method uses marker information from the two parental populations and from the crossbred offspring population. Joint inference of haplotypes is expected to increase accuracy of haplotypes inferred for the three populations. The main objective of our method, however, was to estimate line origin of marker alleles in the crossbred population. The method uses an approach similar to the approach used by Xing et al. [7]. The method can be applied to infer haplotypes and estimate line origin of alleles in crossbred data and to infer haplotypes in purebred data. Throughout this paper, we refer to the method as *DP algorithm *because the algorithm uses a Dirichlet Process as prior distribution for the haplotype frequencies in the parental populations.

The rest of this paper is organized as follows. We begin by describing the DP algorithm, followed by describing the data which we used for evaluating the method. We proceed by describing the results obtained with the method and compare these to results obtained with PHASE [8]. We finish the paper with a discussion section.

## Method

In this section we introduce the DP algorithm for haplotype inference. First, we introduce the concepts involved in the method. Then, we proceed with the details of the method starting with a model for a random mating situation followed by an extension of this model to a situation of crossbreeding. For the implementation of the method, a user can either assume random mating or crossbreeding. We finish the section by describing the evaluation of the method and the data employed in this evaluation. The DP algorithm is programmed in R [17] and available as an R-package upon request from the authors.

### Concepts

Consider a list of genotypes **G **of *L *biallelic loci. The genotype of individual *i*, *G*_*i*_, consists of two unknown haplotypes: the haplotype that the individual received from its mother, *H*_*im*_, and the haplotype that it received from its father, *H*_*if*_. The pair of haplotypes that the individual carries is one of the 2^2*L *^possible haplotype pairs. The probability for each pair is a function of the unknown population frequencies of the two haplotypes.

Imagine that all haplotypes in a population are represented in a list of haplotype classes, **A**, and that a haplotype is identical to the class to which it is associated. Let *c*_*ij *_represent the class in **A **to which haplotype *j *of individual *i *is associated. The associations of all haplotypes in the data to classes in **A **are in matrix **C**. The frequency of a class is the number of haplotypes that are associated to that class.

When genotypes are unordered, neither **A **nor **C **are known. In our method, we need to simultaneously infer the haplotype pair that correspond to a genotype because one haplotype that corresponds to a genotype completely determines the other haplotype corresponding to that genotype.

The length of list **A **represents the haplotype count in the population. When *n *is the number of genotyped individuals and for *n *is greater than 0, this count ranges from 1 to 2*n*. Similar as Xing et al. [7], we formulate the distribution of haplotypes in the population as a mixture model. The mixture components are the elements of **A**. The mixture proportion of a class is proportional to its frequency, which is an estimate of the frequency of that haplotype class in the population.

### Model: random mating situation

We specify a Bayesian model where inference is based on the posterior probabilities of the parameters. The posterior probability of the unknown parameters of our model, **A**, and **C**, is *p*(**A**, **C|G**). Using Bayes' theorem:

(1)

The likelihood of the genotypes given the parameters is *p*(**G**|**A, C**). The prior is *p*(**A**, **C**). We use Gibbs sampling to obtain samples from the marginal posterior distributions of the parameters. For Gibbs sampling, we only need the posterior distribution until proportionality and the normalizing constant *p*(**G**) is not required.

In the following, we describe the likelihood function and the prior distribution for the haplotype classes and the correspondence parameters. We then combine the likelihood and prior and describe our Gibbs sampler.

#### Likelihood function

The following model specifies the likelihood function of our model by describing the relation between genotype *i *and the pair of haplotypes (*H*_*im*_, *H*_*if*_):

(2)

Parameter *q *is an error rate between genotype *i *and pair of haplotypes (*H*_*im*_, *H*_*if*_)*'*. Indicator *I*(*g*_*il *_== *h*_*iml *_+ *h*_*ifl*_) has value 1 when the two alleles at locus *l *match with the genotype on locus *l *and 0 otherwise. Indicator *I*(*g*_*il *_≠ *h*_*iml *_+ *h*_*ifl*_) has value 1 when the two haplotypes do not match with the genotype and 0 otherwise. Because we do not allow for errors, *q *= 1 is in our model. The probability in model 2 is different from 0 only when a pair of haplotypes matches with the genotype on all loci.

#### Prior Distribution

We know that we have a large number *K *of possible haplotype classes (for biallelic loci, *K *= 2^*L*^). For haplotype *j *of individual *i*, *H*_*ij*_, parameter *c*_*ij *_indicates to which class that haplotype is associated. Index *j *∈ (*m, f*)*'*) indicates if the haplotype originated from the mother or from the father of individual *i*. For each class *c*, parameter *ϕ*_*c *_describes the distribution of observations associated to that class and ***ϕ ***represents all *ϕ*_*c *_[18]. For each class, this distribution only consists of haplotypes that are identical to that class because we do not allow for errors between a haplotype and the class to which that haplotype is associated. The *ϕ*_*c *_are sampled from the base distribution of the Dirichlet Process, *G*_0 _[18], which in our case is a distribution the *K *possible haplotype classes. The mixing proportions for the classes, **p**, have a symmetric Dirichlet prior distribution with concentration parameter *α*/*K *[18].

Following Neal [18], this gives:

(3)

The first equation of expression 3 is the distribution of haplotype *H*_*ij *_given parameter *c*_*ij *_and ***ϕ***. The second equation is the prior distribution for *c*_*ij *_= *k*. The third equation is the base distribution of the model and the fourth equation is the prior for the mixing proportions. After integration over **p**, the prior for *c*_*ij *_= *k *is [18]:

(4)

where  is the frequency of haplotype class *A*_*k *_and represents the number of haplotypes associated to this class excluding current haplotype *H*_*ij*_. *n*_*s *_is the number of haplotypes excluding haplotype *H*_*ij*_, i.e. . The first equation is the prior probability of sampling *existing *class *A*_*k*_. The second equation is the prior probability of sampling a *new *class, i.e. the haplotype is not associated to any haplotype class that is already present in list **A**. We modify distribution 3 to evaluate the prior probability of a pair of haplotypes. Here, we integrate the prior for *c*_*im*_, *c*_*if*_|**p **over **p**, because the association of a pair of haplotypes to classes in **A **is unknown. Each haplotype is either associated to an existing class *A*_*k *_in **A **or to a new class which is not in **A**. Five situations can then occur: *a) *Both haplotypes are associated to a different class in **A**; *b) *Both haplotypes are associated to the same class in **A**; *c) *One haplotype is associated to a class in **A **and the other haplotype is associated to a class which not in **A**; *d) *Both haplotypes are associated to different haplotype classes which are not in **A**; *e) *Both haplotypes are associated to the same class which is not in **A**. It can be shown that integration over **p **gives the following prior probabilities for these five situations:

(5a)

(5b)

(5c)

(5d)

(5e)

Here,  represents the number of haplotypes associated to class *A*_*k*_, excluding the two haplotypes corresponding to genotype *i*. The total number of haplotypes sampled excluding the two haplotypes is *n*_*s*_; .

#### Gibbs sampler

We use a Gibbs sampler to obtain samples from the posterior distribution *p*(**c**, **A**|**G**, *q*). We follow algorithm 1 of Neal [18] to derive the posterior probabilities corresponding to the five situations described in the previous:

(6a)

(6b)

(6c)

(6d)

(6e)

The sums in expression 6 can be simplified.  only if *A*_*k *_is compatible with the genotype, i.e. *p*(*G*_*i*_|*c*_*if *_= *A*_*k*_, *q*) = 1. Otherwise it is 0 because one haplotype and a genotype completely determines the second haplotype. To evaluate the sums in the fourth and fifth equation, let *nHet *be the number of heterozygous loci on the genotype. If *nHet *> 0, , otherwise it is 0. If *nHet *= 0, , otherwise it is 0.

Now, conditional expression 6 for the five situations is:

(7a)

(7b)

(7c)

(7d)

(7e)

### Model: crossbred population

We extend the model to a crossbreeding situation. In this situation, we consider three populations. Populations M and F are the purebred parental populations. Population Cross is the crossbred offspring population, created by crossing individuals from population M to individuals of population F. Let **A**_**M **_denote the list of haplotype classes for population M and **A**_**F **_denote the list of haplotype classes for population F. In crossbred individuals, one haplotype originates from population M and the other haplotype originates from population F, and haplotypes inferred for a crossbred genotype thus estimate origin of heterozygous alleles of that genotype. Both haplotypes in a purebred individual from population M or F originate from that population.

**Figure 1:**
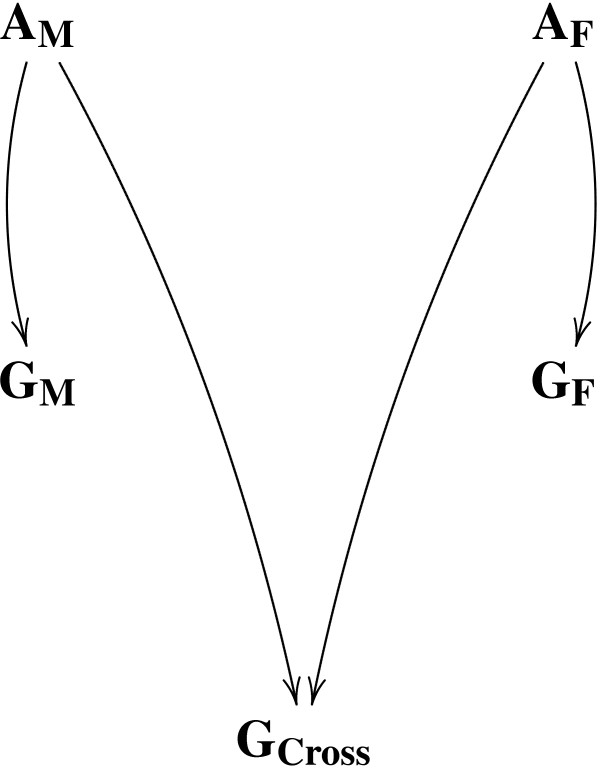
**Graphical representation of the crossbreeding model**. **A**_**M **_represents the list of haplotype classes of population M and **A**_**F **_represents the list of haplotype classes of population F. **G**_**M **_represents the genotypes in population M, **G**_**F **_represents the genotypes in population F, and **G**_**Cross **_represents the genotypes in the crossbred population Cross. Haplotypes for **G**_**Cross **_are associated to classes in **A**_**M **_and **A**_**F**_.

Figure 1 graphically represents this crossbreeding situation with the two list of haplotype classes. Posterior probabilities for sampling haplotype pairs for purebred individuals in population M and F are in expression 7. A different posterior probability is required for sampling a haplotype pair for a crossbred individual.

Haplotype *H*_*im *_of a crossbred individual is associated to a class in **A**_**M **_and haplotype **H**_**if **_is associated to a class in **A**_**F**_. Three situations can occur at the moment of sampling a haplotype pair for a crossbred individual at a given step in the sampling algorithm. *a) *Haplotype *H*_*im *_is associated to a class in **A**_**M **_and haplotype *H*_*if *_is associated to a class in **A**_**F**_. *b) *One haplotype is associated to a class in **A**. and the other haplotype is associated to a class not in the other list of haplotype classes. *c) *Both haplotypes are associated to classes which are not in the lists. The prior probabilities corresponding to these situations are:

(8a)

(8b)

(8c)

The rationale for obtaining posterior probabilities is identical to the single population case. Consequently, the posterior probability for the three situations is:

(9a)

(9b)

(9c)

### Measures of algorithm performance

The goal of our algorithm was to accurately identify line origin of alleles at heterozygous sites in crossbred individuals. For this purpose, the algorithm infers haplotypes for both the purebred and crossbred individuals in the data.

Line origin accuracy of alleles at heterozygous sites in crossbred individuals was assessed using the measure *Allele Origin Accuracy *(*AOAc*). *AOAc *could only be assessed for crossbred individual because all alleles in a purebred individual originate from a single line or population. *AOAc *was calculated as the number of alleles at heterozygous sites whose origin is correctly estimated and is expressed as fraction of the total number of heterozygous loci in that individual. *AOAc *ranges between 0, when origin of all alleles is inferred incorrectly to 1, when origin of all alleles at heterozygous sites is inferred correctly.

For the purpose of estimating allele origin, the algorithm estimates frequencies of haplotype classes in the distinct populations. We used a second measure of algorithm performance to assess the accuracy of inferred haplotype frequencies. Following the article of Excoffier and Slatkin [4], we used *similarity index*, *If*, for this purpose. *If *assesses similarity between the vector of *true *and estimated haplotype frequencies. *If *was calculated as [4]:

(10)

where the summation is over the 2^*L *^possible haplotypes in the population,  is the estimated frequency of haplotype *k *and *p*_*k *_is the true frequency of this haplotype.

We compared *If *of haplotype frequencies inferred with the DP algorithm to *If *of haplotype frequencies inferred with PHASE [8]. We ran PHASE for 1,000 iterations, with a burn-in of 100 iterations and a thinning period of 10 samples, which is the default used by PHASE. *AOAc *could not be compared between the two methods because PHASE assumes single, random mating populations.

Indices *AOAc *and *If *were recorded each 50^*th *^sample of the MCMC chain and averaged over the whole length of the chain to obtain the mean of their posterior distributions. The length of the chain was made dependent on the number of genotypes in the data. For the simulated data, the chain was run for 20,000 iterations when single populations were assumed and for 40,000 iterations when a crossbreeding scheme was assumed. The chain was run for 100,000 iterations for the data of the Wageningen Meishan cross (see below). The first 5,000 iterations were discarded as burn-in. The number of iterations was determined after visual inspection of parameters *If *and *AOAc*, which stabilized after approximately 10,000 iterations.

### Data

We used two datasets to evaluate the algorithm.

#### Simulated data

Two independent populations were simulated (population M and population F). Genomes consisted of one single chromosome of a length of 9 cM with 10 biallelic markers equally distributed over the chromosome. In the base populations, Minor Allele frequencies (MAF) were equal for all markers. In population M, the 1 allele was the minor allele and the 0 allele was the minor allele in population F. For populations M and F, 100 generations of random mating were simulated maintaining a population size of 100 to establish Linkage Disequibrium between markers. Recombinations were simulated according to the genetic distance and without interference. A hundred crossed individuals were simulated by crossing generation 100 of population M to generation 100 of population F. Minor Allele Frequency in the simulated base population was varied between 0.01, 0.25, 0.40, and 0.49 to create a range of situations. In the MAF is 0.49 situation, populations were highly similar, and populations were extremely different in the MAF is 0.01 situation. Ten replicates were simulated for each MAF value.

### Crossbreeding data

The second dataset was SNP data of the Wageningen Meishan-commercial line cross and consisted of 294 genotyped crossbred F1 offspring individuals, 109 genotyped dams from commercial lines, and 19 genotyped sires from the Meishan breed. The genotypes consisted of 14 SNP loci covering approximately 5 cM on chromosome 2. Genotype data of the parental lines (commercial dams and Meishan sires) and genotypes of the crossbred F1 offspring were used for analyses. Haplotypes were previously inferred using the known pedigree with the program CVM (which stands for Cluster Variation Method) [3]. The program CVM is an algorithm for inferring haplotypes from unordered genotype data conditioning on marker information of relatives, identified through pedigree information. Haplotypes inferred with CVM were considered as correct and haplotypes inferred with DP were compared with these.

## Results

In the first part of this section, we validate the algorithm using the simulated data. In the second part, we use the algorithm to estimate haplotypes in the real Wageningen-Meishan cross data. For each dataset, we compare the performance of the DP algorithm with the performance of PHASE.

### Simulated data

Table 1 summarizes the simulated populations. Heterozygosity and the count of distinct haplotypes in the parental population increased when MAF in the base population of M and F increased because MAF was set for reciprocal alleles in the two populations. Chromosome size was equal in all simulations but the number of observable recombinations in the crossbred population increased when MAF of the base population increased because the probability that a haplotype originating from a recombination was already present in the population decreased with increasing heterozygosity.

**Table 1:** Average (standard deviation) of number of distinct haplotypes in (nHap), the average fraction of heterozygous loci within individuals (% het) and fraction observed recombinant haplotypes for the Cross population (% rec).

MAF	Populations M, F		Cross populations		
		
	nHap	% het	nHap	% het	% rec
0.01	2 (1)	0.02 (0.02)	3 (1)	0.98 (0.02)	0.00 (0.00)
0.25	19 (9)	0.20 (0.07)	32 (6)	0.66 (0.08)	0.01 (0.01)
0.40	30 (9)	0.29 (0.06)	50 (12)	0.54 (0.07)	0.02 (0.01)
0.49	32 (8)	0.30 (0.06)	48 (8)	0.49 (0.07)	0.01 (0.01)

nHap and %het in populations M and F represent averages of these two populations. Minor Allele Frequency (MAF) in the base populations was simulated between 0.01 and 0.49. Ten replicates were simulated for each MAF.

The number of haplotype classes increased when concentration parameter *α *of the Dirichlet Process increased (Table 2). There was only a small effect of parameter *α *on *If *of the parental and crossbred populations. Crossbreeding was assumed in these analyses, enabling to calculate *AOAc *for the crossbred population, but the effect of *α *on *AOAc *was only minimal (Table 2).

**Table 2:** Effect of Concentration Parameter (*α*) of the Dirichlet Process on Allele Origin Accuracy (*AOAc*), Similarity Index (*If*), and the average number of haplotype classes () for 1 replicate of populations M and Cross.

	Population M					Cross population				
		
	*α *= 0.01	*α *= 0.1	*α *= 1	*α *= 10	*α *= 100	*α *= 0.01	*α *= 0.1	*α *= 1	*α *= 10	*α *= 100
*AOAc*						0.98	0.98	0.98	0.98	0.97
*If*	0.91	0.91	0.93	0.93	0.92	0.94	0.94	0.94	0.94	0.91
	18	18	18	19	27	47	47	47	49	67

Analyses were run assuming crossbreeding and populations M, F and Cross were used in the analyses. Base populations for M and F were simulated with Minor Allele Frequency equal to 0.40.

Accuracy of estimated haplotype frequencies in the crossbred population was affected by assuming random mating or crossbreeding. When random mating was (erroneously) assumed, there was only 30% agreement between the estimated and true vector of haplotype frequencies in the crossbred population, reflected by *If *(Table 3). *If *increased to 0.87 when crossbreeding was assumed and marker data of the parental populations was included in the analyses (Table 3). Average *If *of haplotype frequencies estimated for the parental M population increased from 0.84 when random mating was assumed to 0.88 when crossbreeding was assumed (Table 3).

**Table 3:** Average (standard deviation) Allele Origin Accuracy (*AOAc*) and Similarity Index (*If*) of haplotypes inferred for genotypes of simulated populations M and Cross.

	Population	*AOAc*	*If*
Random-Mating			
	M		0.84 (0.05)
	Cross		0.30 (0.28)

Crossbreeding			
	M		0.88 (0.03)
	Cross	0.95 (0.02)	0.87 (0.05)

Data were analysed assuming Random-Mating or Crossbreeding. Genotypes of simulated population F were included in the analyses when Crossbreeding was assumed. Analyses were run with *α *equal to 1. Ten replicates where simulated for each scenario. Base populations for M and F were simulated with Minor Allele Frequency equal to 0.40.

Allele Origin Accuracy was only calculated for crossbred individuals when crossbreeding was assumed. In this case, *AOAc *was 0.95, reflecting that the origin of 95% of the alleles at heterozygous sites in crossbred individuals was correctly assessed.

Including marker data of at least one parental population was crucial for *AOAc *and *If *of haplotypes inferred for crossbred individuals (Table 4). A lower improvement was achieved due to including the second population in the analyses.

**Table 4:** Average Allele Origin Accuracy (*AOAc*) and Similarity Index (*If*) of haplotypes inferred for genotypes of simulated Cross population.

	*AOAc*	*If*
100% Pop. M, F	0.95 (0.02)	0.87 (0.05)
100% Pop. M, 0% Pop. F	0.94 (0.01)	0.84 (0.03)
0% Pop. M, F	0.44 (0.19)	0.36 (0.21)

Analyses were run assuming Crossbreeding and purebred populations M and F were either included or not in the analyses. Analyses were run with *α *equal to 1. Populations were simulated with Minor Allele Frequency in the base populations equal to 0.40. Ten replicates were simulated for each scenario.

Similarity Index and *AOAc *of haplotypes inferred for crossbred individuals with DP increased when MAF of the parental populations were increasingly different (Table 5). In contrast, *If *of haplotypes inferred for the same data with PHASE decreased when differences between MAF of parental populations increased (Table 5). *If *of haplotypes inferred for purebred individuals were similar between DP and PHASE.

**Table 5:** Average (standard deviation) of Similarity Indices *If *for haplotypes inferred with PHASE and with the DP algorithm from genotypes of simulated populations M and Cross.

MAF	PHASE		DP	
		
	Pop. M	Cross pop.	Pop. M	Cross pop.
0.01	1.00 (0.01)	0.00 (0.00)	1.00 (0.01)	1.00 (0.01)
0.25	0.93 (0.04)	0.12 (0.28)	0.93 (0.04)	0.92 (0.04)
0.40	0.86 (0.05)	0.42 (0.30)	0.88 (0.03)	0.87 (0.05)
0.49	0.90 (0.03)	0.55 (0.25)	0.90 (0.03)	0.89 (0.03)

Minor Allele Frequency in the base populations (MAF) was simulated between 0.01 and 0.49, 10 replicates were simulated for each MAF. Genotypes of simulated population F were included in the analyses with the DP algorithm. Parameter *α *was set equal to 1 in the analyses with DP.

### Wageningen Meishan-Commercial cross

The crossbred individuals in the Wageningen Meishan-Commercial cross data originated from 19 sires and 109 dams. Three analyses were performed using data of 19, 63 and 109 dams and only their offspring and the sires of these offspring in the analyses. Data were analysed using the DP algorithm assuming crossbreeding, using the DP algorithm assuming random mating and using PHASE, which assumes random mating.

Similarity Indices obtained using the DP algorithm were substantially higher when crossbreeding was assumed compared to when random mating was assumed (Table 6). Similarity indices obtained with PHASE were very similar to *If *obtained with DP assuming crossbreeding, despite that PHASE assumed random mating. There was not a clear effect of the number of dams used on *If*.

**Table 6:** Allele OriginAccuracy (*AOAc*) and Similarity Index (*If*) for haplotypes inferred with the DP algorithm assuming crossbreeding (DP), with the DP algorithm assuming random mating (DP RM) and with PHASE.

	DP CB		DP RM	PHASE
			
	AOAc	If	If	If
19 Dams				
Cross	0.97	0.93	0.09	0.93
Dams		0.92	0.90	0.86
Sires		0.75	0.78	0.80

63 Dams				
Cross	0.94	0.87	0.69	0.86
Dams		0.84	0.80	0.83
Sires		0.76	0.77	0.77

109 Dams				
Cross	0.95	0.91	0.10	0.91
Dams		0.84	0.82	0.81
Sires		0.76	0.77	0.77

Parameter *α *of the DP algorithm was set equal to 1. Data from the Commercial × Meishan crossbreeding data. Indivuals in the Dam group were from the commercial breed and individuals in the Sire group were from the Meishan breed. Parameter *α *was set equal to 1 in the analyses with DP.

Allele origin accuracies obtained with DP when crossbreeding was assumed were approximately 0.95, without regard of the number of dams included in the data (Table 6).

## Discussion

Crossbreeding or hybridisation is a common case of nonrandom mating in animal and in plant breeding. Inference of haplotypes in crossbred individuals is useful when line origin of alleles is required because haplotypes provide information about cosegregation of chromosome segments. In this paper, we introduced and validated a method for estimating line origin of alleles in crossbred individuals when pedigree information is unknown.

To our knowledge, no algorithms for estimating line origin of alleles in crossbred individuals have been described. Comparison of results obtained with the DP algorithm to results obtained with alternative methods was therefore not possible. For comparison, we concentrated on the accuracy of haplotype frequencies, as indexed by parameter Similarity Index, *If *and compared *If *obtained using the DP algorithm to *If *obtained using PHASE.

PHASE was used to compare results obtained with the DP algorithm because PHASE was used in several recent studies (e.g. [19-21]). The prior distribution for haplotypes used in PHASE is more realistic than that used in the DP algorithm. The prior distribution in the DP algorithm assigns equal probability to all classes from the 2^*L *^possible haplotypes. The prior distribution in PHASE approximates a coancestry model of the haplotypes and explicitly models linkage between markers [1,8]. Haplotypes inferred with PHASE for the Wageningen Meishan-Commercial cross data reflect the qualities of PHASE (Table 6). In the situations which were simulated, however, haplotypes for crossbred individuals inferred with PHASE were less accurate than haplotypes inferred with DP.

Complexity of haplotype inference is determined by the number of heterozygous loci in a genotype because the number of possible haplotype configurations is 2^*nHet*^. By design of the simulations, heterozygosity in the crossbred populations was high when heterozygosity in the parental populations was low (Table 1). Consequently, *If *of haplotype frequencies inferred with PHASE where low for the crossbred populations and high for the parental populations in these scenarios (Table 5). In contrast to PHASE, the DP algorithm uses information from the two parental populations to infer haplotypes in the crossbred population. Advantage of this approach was most apparent in situations when *If *of haplotypes inferred with PHASE for crossbred individuals were lowest.

Line origin of approximately 95% of the alleles at heterozygous sites in crossbred individuals was correctly identified by the algorithm when genotypes of parental individuals were included in the analyses. Excluding genotypes of either one or both parental populations from the analyses showed that including data of at least one parental population was crucial for correct identification of line origin of alleles (Table 3).

In the current DP algorithm, the prior distribution for haplotype classes does not account for allele frequencies in each population. Clustering haplotypes based on allele frequencies, following Huelsenbeck and Andolfatto [22], could improve the accuracy of the DP algorithm for crossbred individuals, especially in situations when few data on the parental populations are available. In addition, it could facilitate extension of the algorithm to situations where the data originated from more than two parental populations. Currently, the algorithm can not easily be extended to more than two population because of the large number of possible haplotype configurations which would need to be evaluated for this because each haplotype could originate from all populations.

The DP algorithm is similar to the algorithm of Xing et al. [7] because it assumes the existence of a limited number of classes for the haplotypes in the population and uses a Dirichlet Process as prior distribution for these classes. A feature of the Dirichlet Process is that it clusters data without the need to specify the number of clusters. In the context of haplotypes, this feature is especially attractive because the haplotype diversity in the population usually is lower than the 2^*L *^possible haplotype classes (*L *is the number of polymorphic loci in the data).

Apart from the ability to infer haplotypes in a situation of crossbreeding, the most important difference between our model and that of Xing et al. [7] is that our model does not assume errors between a haplotype and the class to which it is associated nor between a pair of haplotypes and the genotype to which they correspond. The first consequence of this is that we need to update the pair of haplotypes corresponding to a genotype simultaneously because the haplotypes corresponding to a genotype are conditionally dependent. The second consequence is that the number of haplotype classes required for a population is equal or larger than in the model of Xing et al. [7].

Not not allowing for errors had several benefits. Implementation of the model of Xing et al. [7] showed that controlling the error rate through the hyperparameters of their model was very difficult. Errors were either sampled between haplotypes and their classes or between haplotypes and the genotypes to which they corresponded. Not allowing for errors between haplotypes and genotypes made simultaneously updating the pair of haplotype corresponding to a genotype necessary. For simultaneous updating, however, all pairs of haplotypes that are possible for a genotype need to be considered in each sampling step of the algorithm. Not allowing for errors between haplotypes and the classes to which they correspond is then advantageous because it reduces the number of possible haplotype pairs for a genotype from 2^2*L *^to 2^*nHet *^(*nHet *is the number of heterozygous loci at a genotype).

The number of markers used in both the simulated and the real data is low compared to number of markers that are currently used. Two problems are expected when the number of markers in the data increases. The first and most trivial one is the size of the data which obviously increases. The second problem is that haplotypes become increasingly unique when markers are located on regions more distant on the genome due to occurrence of recombinations and random sampling of independent chromosomes. Performance of the DP algorithm can be expected to be low when the number of haplotypes unique in the crossbred population increases. A practical solution could be to split the data into subsets of adjacent markers on single chromosomes or to use a sliding window approach over chromosomes.

The algorithm could be adapted to allow for missing marker data. Let *m *be the number of missing markers for a specific individual. The likelihood in Expression 2 should then only be evaluated for the *L *- *m *non missing markers, since the other markers always match. The summations in Expressions 6, 7 and 9 should only account for the number of non missing markers, *L *- *m*. In essence, the model would need to evaluate the non missing markers in each individual, since individuals are sampled independently.

In the present article, we introduced a new algorithm for inference of line origin of alleles in crossbred populations. Analyses with both simulated and real data showed that origin of approximately 95% of the alleles at heterozygous sites was inferred correctly. Application of the algorithm to realistic data will require extension of the algorithm with methods to deal with large numbers of markers and with missing data.

## Competing interests

The authors declare that they have no competing interests.

## Authors' contributions

JD and RF drafted the initial questions. RF and AC developped the statistical methods. AC drafted the manuscript and wrote the software. HH supervised the work of AC. JD, RF and HH critically reviewed the manuscript. All authors read and approved the manuscript.
